# Involvement of Neuroinflammation and Oxidative Stress in L-DOPA-Induced Dyskinesia in Parkinson’s Disease: Role of Renin–Angiotensin System and ROCK Pathway

**DOI:** 10.3390/antiox14101154

**Published:** 2025-09-23

**Authors:** Ana Muñoz, Andrea López-López, Jannette Rodríguez-Pallares, José Luis Labandeira-Garcia

**Affiliations:** 1 Research Center for Molecular Medicine and Chronic Diseases (CiMUS), Health Research Institute of Santiago de Compostela (IDIS), Universidade de Santiago de Compostela, 15782 Santiago de Compostela, Spain; andrealopez.lopez@usc.es (A.L.-L.); jannette.rodriguez@usc.es (J.R.-P.); joseluis.labandeira@usc.es (J.L.L.-G.); 2 Networking Research Center on Neurodegenerative Diseases (CIBERNED), 28029 Madrid, Spain

**Keywords:** levodopa, non-neuronal mechanism, glial cells, cytokines, interleukin-1β, candesartan, fasudil, ROS

## Abstract

Dopamine (DA) replacement by L-DOPA administration is the most common and effective treatment for Parkinson’s disease (PD). However, its chronic use leads to important side effects at advanced stages of the disease. Levodopa-induced dyskinesia (LID), characterized by involuntary, abnormal movements, is the main challenge of L-DOPA treatment. Although the causes underlying LID are not fully understood, abnormal plasticity in corticostriatal synapses and dysregulated DA release from serotonin terminals play a crucial role. In recent years, several studies have suggested the involvement of neuroinflammation and oxidative stress in the pathophysiology of LID. Interestingly, different evidence has shown that blocking these pathways reduces LID in experimental animal PD models, pointing to the use of antioxidant/anti-inflammatory agents as a potential therapy for LID. Numerous studies have shown the role of the brain renin–angiotensin system (RAS) and the ROCK pathway in neuroinflammation and oxidative stress. Compounds acting through these routes have strong neuroprotective properties in PD models. Additionally, the use of ROCK inhibitors, such as fasudil, and RAS blockers has shown potent anti-dyskinetic effects. Therefore, compounds acting on the RAS and ROCK pathways could have a dual role, slowing down the degeneration of dopaminergic neurons and reducing the development of LID.

## 1. Introduction

Parkinson’s disease (PD) is the second most common neurodegenerative disease affecting millions of people in the world. In the last few years, PD incidence has increased significantly due to population ageing, resulting in serious health, social, and economic problems [1,2]. PD is characterized by a selective loss of dopaminergic neurons located in the substantia nigra, resulting in decreased dopamine (DA) levels in the striatum and alterations in the basal ganglia circuits with a crucial role in movement control [3]. Therefore, the main features of the disease are motor symptoms, leading to bradykinesia, rigidity, gait abnormality, or resting tremors. However, non-motor deficits like anosmia, REM sleep behavior disorders, or gut disturbances often appear earlier in the disease. These signs, combined with several biomarkers, can be useful for an early diagnosis of the disease [4,5,6]. Environmental and genetic factors influence PD risk, but the mechanisms involved in the pathogenesis of the disease are not fully understood. Nevertheless, mitochondrial dysfunction, oxidative stress, protein aggregation, impaired autophagy, autoimmune processes, and neuroinflammation are involved in the neurodegeneration of dopaminergic neurons, a key feature of the disease [7].

The DA precursor L-3,4-dihydroxyphenylalanine (L-DOPA), administered in conjunction with carbidopa, is the most used form of therapy for PD and remains the most successful treatment currently [8]. Unfortunately, its chronic use leads to important side effects in advanced stages of the disease, limiting the therapeutic effect of this treatment. L-DOPA-induced dyskinesia (LID) is the main challenge of L-DOPA treatment, consisting of the development of involuntary abnormal movements produced by DA fluctuations, with a significant decrease in the quality of life of PD patients [9]. LID is characterized by abnormal involuntary movements, such as stereotypical, choreiform, and throwing movements, as well as head, neck, trunk, and limb dystonia [9,10,11]. It has been estimated that up to 80% of patients suffer from LID after an average of 6.5 years of treatment [10,11,12]. Several risk factors are involved in the appearance of dyskinesia, in particular, young age at PD onset, the duration and severity of Parkinsonian motor symptoms, and a higher cumulative L-DOPA dosage [13]. However, the severity of striatal DA denervation is a major risk factor for the development of LID.

**Figure 1:**
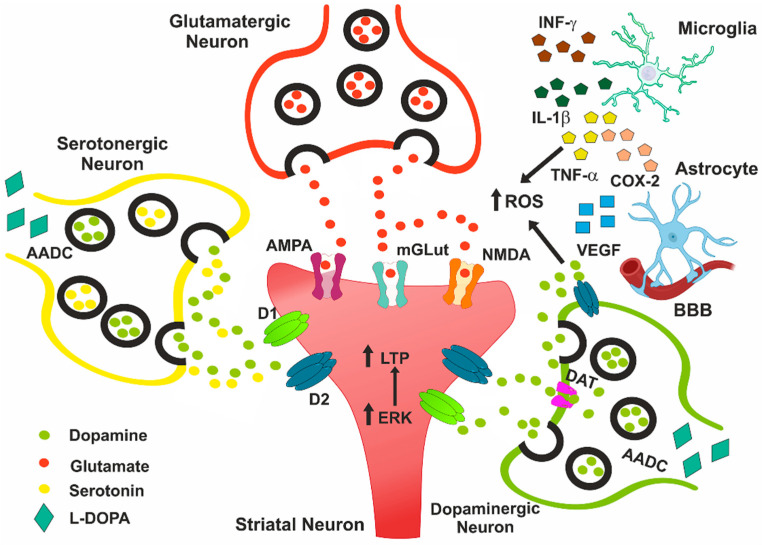
Mechanisms involved in the development of L-DOPA-induced dyskinesia (LID). Overactivity of glutamatergic corticostriatal neurons and the non-regulated release of dopamine (DA) by the serotonergic neurons trigger LID. However, non-neuronal mechanisms, such as cytokines produced by glial cells, are also involved, affecting the synaptic plasticity. An increase in reactive oxygen species (ROS), promoted by pro-inflammatory cytokines and the excess of DA, also contributes to LID. AADC, L-amino acid decarboxylase; AMPA, alpha-amino-3-hydroxy-5-methyl-4-isoxazolepropionic acid receptor; BBB, blood–brain barrier; COX-2, cyclooxygenase-2; D1, D1 dopamine receptor; D2, D2 dopamine receptor; DAT, dopamine transporter; ERK, extracellular-regulated kinase; IFN-γ, interferon-gamma; IL-1β, interleukin-1 beta; LTP, long-term depression; mGlut, metabotropic glutamate receptor; NMDA, N-methyl-D-aspartate glutamate receptor; TNF-α, tumor necrosis factor-alpha; VEGF, vascular endothelial growth factor. Created in BioRender. https://BioRender.com/h42n006, accessed on 11 September 2025.

Dyskinesias are associated with plasma L-DOPA concentration fluctuations resulting in intermittent stimulation of D1-type and D2-type DA receptors. Although the causes of LID are not fully understood, the use of animal PD models, such as the infusion of 6-hydroxydopamine (6-OHDA) in rodents as the classical model, has allowed the discovery of several mechanisms [14,15,16] (see Figure 1).

First, the overactivation of glutamatergic synapses and abnormal synaptic plasticity are crucial in the onset of LID, as a post-synaptic mechanism [17] (see for review [18]) (see Figure 1). Indeed, amantadine, a low-affinity, non-competitive NMDA receptor antagonist, is commonly used in clinical settings for its moderate anti-dyskinetic effects. However, its use is often associated with adverse effects [19]. Recent studies showed that manipulation of the glutamatergic system, employing strategies such as activation of mGlut 2/3 receptors [20], striatal *GluN2A* gene suppression [21], or optogenetic approaches [22], alleviates dyskinesia. Compounds that are capable of modulating the activity of the glutamatergic system, such as safinamide, ameliorated motor deficits and delayed the onset of LIDs in preclinical models, and their effect in PD patients has been explored [23,24] (JapicCTI-153057). Aberrant plasticity of the cortico-basal ganglia is related to changes in LTP (long-term potentiation) and LTD (long-term depression), the main mechanisms of synaptic plasticity in the brain [17,18] (see Figure 1).

Second, the serotonin system emerged as a key element in the development of dyskinesia, as a presynaptic mechanism [25]. Thus, in advanced stages of the disease, when most of the dopaminergic neurons have degenerated, L-DOPA is converted to DA in the serotonergic terminals, which mediates DA release as a “false” transmitter [26,27,28]. The lack of autoregulatory feedback control in serotonergic neurons to control L-DOPA-derived dopamine release contributes to the development of LID [25] (see Figure 1). Consistent with this, the administration of agonists targeting serotonergic autoreceptors, such as buspirone or sarizotan [29,30,31,32], as well as chemogenetic inhibition of serotonin raphe-projecting neurons [33], has been shown to suppress LID in both rodent and primate models. These interventions have also demonstrated moderate efficacy in patients with PD [34]. Moreover, serotonergic regulation of synaptic DA levels through the administration of serotonin transporter inhibitors such as citalopram, or the serotonin precursor 5-hydroxy-tryptophan (5-HTP) [35], or simultaneous 5-HT1B receptor agonist with the 5-HT6 receptor antagonist [36], reduces LID in preclinical models. Although the role of postsynaptic and presynaptic mechanisms in the development of LID has been debated in recent years, most researchers now support a multifactorial model, in which LID arises from the interaction between dysregulated DA release (presynaptic) and aberrant plasticity and signaling in striatal neurons (postsynaptic). Evidence suggests that a feedback loop exists in which presynaptic dysregulation leads to abnormal postsynaptic plasticity, potentially regulating further pre-/post-interactions [11]. It has been described that other pathways involving adenosine receptors are also involved in the development of dyskinesia, presenting itself as a valuable therapeutic target. These receptors are found at both presynaptic (in dopaminergic and glutamatergic terminals) and postsynaptic levels in the striatum. Recently, it has been shown that the adenosine A2A receptor antagonist/inverse agonist KW-6356 can be used to potentiate the effects of a wide range of L-DOPA doses, with a low risk of dyskinesia for the treatment of PD [37].

**Figure 2:**
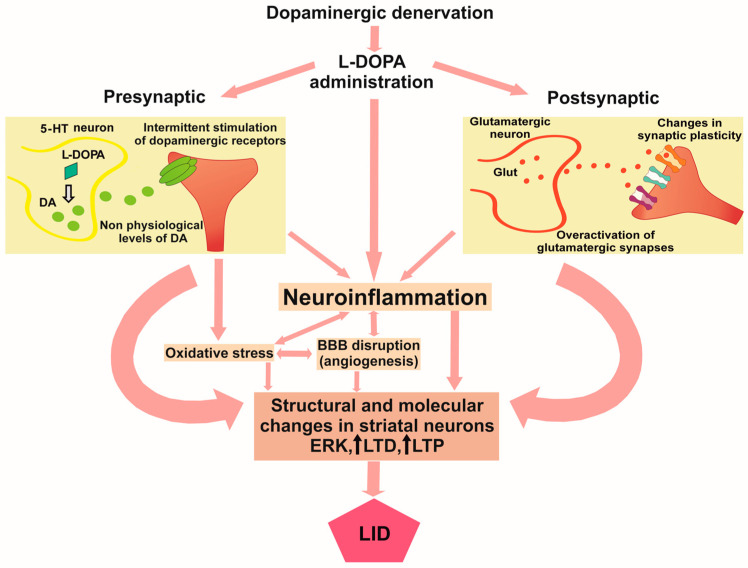
Conceptual framework shows the interactions between the mechanisms involved in LID. In PD patients with DA denervation, L-DOPA administration leads to overactivity of corticostriatal synapses as a postsynaptic mechanism, together with a non-regulated release of DA by the serotonergic terminals, as a presynaptic mechanism. Non-physiological levels of DA cause intermittent stimulation of DA receptors, neuroinflammation, oxidative stress, and BBB disruption, leading to structural and molecular changes in striatal neurons producing LID. 5-HT, serotonin; DA, dopamine; Glut, glutamate; BBB, blood–brain barrier; ERK, extracellular-regulated kinase; LTP, long-term depression; LTP, long-term potentiation; LID, L-DOPA-induced dyskinesia.

Lastly, it is noteworthy that in recent years, non-neuronal mechanisms related to neuroinflammation, angiogenesis, and oxidative stress have also been suggested in the pathophysiology of LID [38] (see Figure 2). This has led to the identification of new therapeutic targets for managing LID. Indeed, blocking these pathways has been shown to reduce dyskinesias [39,40,41,42]. Oxidative stress and neuroinflammation are key mechanisms in the pathophysiology of PD and the degeneration of dopaminergic neurons. Therefore, compounds acting through these pathways, in addition to reducing dyskinesias, would have neuroprotective effects that would slow the progression of PD. Interestingly, previous studies showed evidence that the brain renin–angiotensin system (RAS) and the Rho kinase (ROCK) pathway modulate neuroinflammation and oxidative stress in PD, pointing to these systems as possible new targets for the treatment of LID [39,41,43,44,45,46,47,48]. In this review, we summarize the findings about the role of neuroinflammation and oxidative stress in LID, highlighting new and promising therapeutic strategies for managing LID and PD through emerging targets involved in neuroinflammatory and oxidative stress pathways.

## 2. Role of Oxidative Stress in Pathophysiology of LID

An adverse effect of L-DOPA treatment may involve the induction of free radical-mediated oxidative stress, which can facilitate the development of hyperkinetic movements [49]. The increase in oxidative stress observed in LID could be caused by excessive DA concentrations and metabolism, as L-DOPA, the precursor of DA, promotes the production of free radicals. Consistent with this, several studies have demonstrated that various antioxidant compounds can reduce LID in animal models. The α-lipoic acid, an antioxidant naturally synthesized in the human body with potential therapeutic value, reduces LID in 6-OHDA Parkinsonian rats without compromising the anti-Parkinsonian effect of L-DOPA. This compound decreased malondialdehyde (MDA) and glutathione (GSH) levels, reduced the number of ionised calcium binding adaptor molecule (Iba-1)-positive microglial cells, and overexpressed cleaved-caspase-3 in the substantia nigra [50]. Remarkably, it has been reported that α-lipoic acid can also protect dopaminergic neurons against 1-methyl-4-phenyl-1,2,3,6-tetrahydropyridine (MPTP)-induced apoptosis by attenuating reactive oxygen species (ROS) formation [51,52]. It is also interesting to note the effect of resveratrol, the most well-known polyphenolic stilbenoid, found in grapes, peanuts, mulberries, and several other plants. Resveratrol has antioxidant, anti-aging, anti-inflammatory, anti-carcinogenic, and anti-microbial properties, and attenuates dyskinesia without the compromising anti-Parkinsonian effects of L-DOPA. In addition, resveratrol reduces the protein expression of dyskinesia-related molecular markers, including ΔFOS B, and extracellular regulated kinase (ERK) in the striatum and inhibits microglia and astroglia activation in the substantia nigra, thereby preventing subsequent inflammatory responses in the striatum [42]. Interestingly, resveratrol showed neuroprotective properties in PD [53].

Recently, it has been shown that other compounds with antioxidant properties could reduce LID. Liquiritigenin-rich hydroalcoholic extract of Brazilian red propolis, a natural product with a high flavonoid content and known for its antioxidant and anti-inflammatory properties, reduces dyskinesia in combination with amantadine, and these effects may be mediated by astrocyte-related mechanisms [54]. Moreover, polysaccharides extracted from the rhizomes of *Polygonatum cyrtonema* alleviated dopaminergic neuron apoptosis and dyskinesia in a PD mouse model by inhibiting oxidative stress and endoplasmic reticulum stress [55]. Meanwhile, a recent study showed that PD patients with LID showed a significant reduction in antioxidant activity, together with an upregulation of inflammatory markers such as interleukin-1β (IL-1β), TOLLIP (a protein with a key role in inflammatory signaling, autophagy, and the transport of vacuoles within the cell), and C-reactive protein levels [56]. Other findings suggest that nitric oxide (NO) is involved in the development of LID through a post-synaptic mechanism, reducing the L-DOPA-induced increase in ΔFosB, phospho-DARPP-32, and phospho-GluA1 α-amino-3-hydroxy-5-methyl-4-isoxazolepropionic acid (AMPA) receptor subunit levels [57,58]. It is also suggested that nitric oxide synthase (NOS) inhibitors attenuate the development of LID [58,59,60]. Recently, it has been shown that LID is associated with depletion of antioxidant plasmalogen phosphatidylcholines in regions critical for motor function, along with elevations in polyunsaturated fatty acid-containing glycerophospholipids. This suggests that lipid composition mediates differential susceptibility to LID since lipid alterations correlated strongly with dyskinesia severity, DA, and L-DOPA concentrations, supporting a mechanistic link between lipid metabolism, neurotransmitter dysregulation, and LID [61].

Other molecular pathways that could be explored in LID related to oxidative stress include key enzymes in the generation of ROS in neurons, where NADPH oxidase plays a prominent role. There are several isoforms of NADPH oxidase (NOX1, NOX2, NOX3, NOX4, NOX5, DUOX1, and DUOX2), collectively known as the NOX system. The most important isoforms in the brain are NOX2 (gp91phox) and NOX4 [62,63,64]. NOX2 contributes to progressive oxidative damage, which, in turn, can lead to α-synuclein accumulation and mitochondrial protein impairment. Moreover, NOX2 inhibitors hold potential as a disease-modifying therapy in PD [62]. NOX4 has been proposed as a biomarker and therapeutic target in neurodegenerative diseases [65]. NADPH activation increases dopaminergic degeneration by ferroptosis and neuroinflammation. Conversely, inhibition of NOX4 reduces lipid peroxidation and iron accumulation in PD animal models, accompanied by improvements in behavioral impairments [66]. Mitochondrial complex I is another source of ROS and oxidative stress. It has been reported that the increased oxidative stress reported in patients treated with L-DOPA may result from reduced levels of antioxidants, impaired mitochondrial transport, and excessive oxidation of DA [67]. Moreover, DA toxicity involves mitochondrial complex I inhibition [68]. Curiously, several studies have suggested an inherent vulnerability of the mitochondria in the putamen in some PD patients, rendering them more susceptible to developing dyskinesias. Other PD patients seem to have a higher resilience to mitochondrial damage, which could prolong the time to manifestation of dyskinesias. Reduced levels of mitochondrial DNA and increased mitochondrial DNA deletions observed in dyskinesia–PD patients indicate an underlying mitochondrial pathology, potentially involving mitochondrial complex I [69]. Accordingly, mitochondrial function could represent an additional therapeutic target for treating LID. A recent study also revealed that cerebral iron deposition may be involved in LID development, and oxidative stress, neuroinflammation, and mitochondrial and lysosomal dysfunction play a critical role [70]. Further studies would be necessary to elucidate the interaction between these factors and the mechanisms through which oxidative stress is involved in LID. Whether oxidative stress is a cause or a consequence of abnormal dopaminergic signaling in LID has not been fully proven. It has been suggested that excess of L-DOPA and DA increases ROS and free radicals, which contribute to the induction of LID [42], suggesting that chronic use of L-DOPA induces systemic or peripheral oxidative stress as a consequence [56]. However, increased neuronal activity associated with involuntary movements could increase mitochondrial activity, which, in turn, generates higher levels of ROS and free radicals. Overall, several antioxidant compounds have shown anti-dyskinetic properties and exert their effects by reducing neuroinflammation, which has recently been proposed as a key mechanism involved in the development of LID.

## 3. Role of Neuroinflammation in LID

Over the last few years, neuroinflammation has been linked to LID, suggesting a role in the adverse effects of L-DOPA treatment [38,71,72,73]. Numerous preclinical studies, most of them using the 6-OHDA rat model, confirmed an inflammatory pathology in LID, showing glial activation [59] related to increased immunoreactivity of striatal OX-42-microglial cells, glial fibrillary acidic protein (GFAP)-positive astrocytes, and increased levels of tumor necrosis factor-alpha (TNF-α), inducible NOS (iNOS) [49], Ile-1β [74,75], and interferon-gamma (IFN-γ) [76]. Different studies using the MPTP primate model reported an inflammatory response in dyskinetic monkeys, showing increased levels of Iba-1, CD68, and GFAP [77]. These results were also confirmed in studies in patients with LID, where high levels of IL-1β were shown [56]. Moreover, these PD patients showed a distinct metabolic profile associated with LID, both in plasma and cerebrospinal fluid (CSF), which may be related to the dysregulation of lipid metabolism and neuroinflammation [78]. Furthermore, systemic inflammation has been shown to exacerbate pre-existing neuroinflammation, creating a vicious cycle that amplifies inflammatory response and contributes to the development of LID [79].

The inflammatory response associated with LID can be attributed to several mechanisms. Astrocytes are increasingly recognized as playing a remarkable role in developing LID (see Figure 1). Different studies suggest that astrocytes transport L-DOPA from the blood to the brain and take up L-DOPA through an amino acid transporter. Moreover, astrocytes express DA transporters, and the monoamine oxidase B (MAO-B) and catechol-O-methyltransferase (COMT) enzymes, which participate in L-DOPA metabolism, and the enzyme aromatic L-amino acid decarboxylase (AADC), essential to convert L-DOPA to DA [73,80]. In a recent study, using chemogenetic tools in GFAP-expressing cells, the authors showed that modulating astrocytic activity in the striatum mitigates LID [81]. Interactions between astrocytes and dopaminergic neurons are complex; however, it is known that L-DOPA administration can induce astrocytic hyperactivation. Consequently, astrocytes may contribute to striatal dysfunction and produce toxic molecules, including NO and cytokines [59,73]. Astrocytes also express DA receptors, with the DA acting as a modulator of astrocyte-to-neuron communication. Astrocytic D3 receptor has been proposed to play a controversial role in inflammation and astrogliosis [82]. Interestingly, D3 receptor is overexpressed in the striatum of dyskinetic animals, and blockade of this receptor reduces the severity of LID [83].

Microglial cells are also involved in LID development. Chronic use of L-DOPA activates microglia, leading to an inflammatory response in the striatum and the release of cytokines. This alters synaptic plasticity, leading to changes in LTP and LTD, thereby promoting the development of dyskinesias [12] (see Figure 1). In fact, increased levels of proinflammatory cytokines secreted by microglia can affect glutamatergic transmission and corticostriatal plasticity, mechanisms linked to dyskinesia [80]. Accordingly, Morissette and colleagues showed that blocking metabotropic glutamate receptors prevents LID, reducing inflammation in the brain of Parkinsonian monkeys [77]. Transcriptome analysis revealed an important role of IL-1β, TNF-α, transforming growth factor-beta (TFG-β), and IFN-γ in the development of LID [84,85]. In line with this, abnormal levels of these cytokines were found in the CSF and tissues of PD patients [84].

### 3.1. Interleukin-1β

Our group and others have shown a significant increase in IL-1β levels in dyskinetic rats compared with non-dyskinetic animals [40,41,45,46,86]. Both in experimental animal models and in PD patients, IL-1β levels are upregulated, and this is related to aging-derived neuroinflammation [87,88]. This finding is consistent with the higher risk of developing LID in aged subjects. In an interesting study conducted by Lanza and collaborators [75], aging-associated exacerbation of LID was correlated with neuroinflammation in the hemi-Parkinsonian rat model. Their results showed that aged rats (i.e., 18-month-old rats) treated acutely with L-DOPA exhibited higher extracellular concentrations of IL-1β, whereas control rats showed higher extracellular IL-6 levels. Interestingly, administration of an IL-1β antagonist reduced LID in this rat model [74].

### 3.2. Tumor Necrosis Factor-Alpha

Different studies support the involvement TNF-α in the pathophysiology of LID. TNF-α plays an important neuromodulatory role in the brain, regulating neurotransmitter metabolism and synaptic and brain plasticity. High levels of TNF-α are detected in the brain and CSF of PD patients, suggesting their involvement in the neuroinflammation associated with the disease [89]. Moreover, preclinical studies have shown that a glial response in the striatum of Parkinsonian animals developing dyskinetic responses following L-DOPA administration is associated with increased levels of TNF-α and IL-1β [40,86], see for review [90]. To investigate the mechanisms of neuroinflammatory processes in LID, astrocytes and microglial cells in vitro were exposed to L-DOPA, DA, or glutamate, as the candidate molecules that might operate as inflammatory cues during LID development. Neither L-DOPA nor DA produced an inflammatory response in glial cell cultures. However, glutamate enhanced TNF-α secretion and GFAP expression in astrocytes and increased Iba-1 expression in microglial cells, suggesting that the release of TNF-α by glutamate-activated astrocytes may contribute to LID by exacerbating corticostriatal glutamatergic inputs, thereby maintaining astrocytes in an activated state [86]. Furthermore, other evidence suggests that TNF-α may contribute to the altered neuronal responses in LID by targeting receptor trafficking and function in striatal neurons, synaptic plasticity, DA synthesis in preserved dopaminergic terminals, and serotonin metabolism in serotonergic neurons [72].

### 3.3. Interferon-Gamma

IFN-γ is another key player involved in neuroinflammatory processes. This proinflammatory cytokine activates glial cells and is increased in the plasma and brain of PD patients [91]. In an insightful study, Ferrari and colleagues [76] investigated whether IFN-γ deficiency could influence nigrostriatal degeneration, LID, and neuroinflammation. They used wild-type (WT) and IFN-γ-knockout (KO) mice for their experiments. Although no significant differences in dyskinesia severity were observed between WT and IFN-γ KO mice, IFN-γ KO mice treated with L-DOPA showed increased levels of iNOS and GFAP staining in the 6-OHDA-lesioned striatum compared with the WT group.

IFN-γ activates the transcription factor NF-κB, which plays a key role in immune regulation and inflammatory responses. This factor may also contribute to the development of LID. Indeed, agmatine, a product of L-arginine decarboxylation, has been shown to reduce dyskinesia in rotenone-lesioned rats by inhibiting inflammatory and oxidative stress pathways, including suppression of the NF-κB signaling cascade [92] (see Table 1).

### 3.4. Cyclooxygenase-2

Cyclooxygenase-2 (COX-2) is an enzyme involved in the formation of prostaglandins, such as prostaglandin (PG) E2, key mediators of the inflammatory response and pain. COX-2 inhibitors are widely used to reduce inflammation. COX-2 also contributes to inflammatory pathways in neurodegeneration and plays a role in the progression of PD [99]. Parkinsonian dyskinetic rats, after receiving L-DOPA, showed increased neuronal COX-2 immunoreactivity, which directly correlated with dyskinesia severity. COX-2 is also increased in the striatum of dyskinetic mice, but its role as a causal factor or consequence remains unclear. Moreover, inhibition of NOS prevented LID and the increase in COX-2 expression in the dorsal striatum, the preferential region involved in LID [100]. Recently, COX-2 expression was associated with LID development after 14 days of L-DOPA treatment, correlating with LID severity and FosB/ΔFosB expression, as molecular markers of dyskinesia. However, COX-2 inhibition did not reduce dyskinesia or COX-2 and FosB/ΔFosB expression, while significantly lowering PGE2 levels and oxidative stress in mouse and primate PD models [95]. Meanwhile, the same authors showed that doxycycline, a semisynthetic antibiotic, reduced LID accompanied by decreased Fos-B and COX-2 expression and reduced levels of PGE2, TNF-α, and IL-1β in the dorsolateral striatum of dyskinetic mice [96]. Overall, these results point to COX-2 as a relevant factor in the development of dyskinesia.

### 3.5. Vascular Endothelial Growth Factor

Angiogenesis also plays a role in LID associated with neuroinflammation. Both animal models of LID and PD patients exhibit increased levels of vascular endothelial growth factor (VEGF) alongside inflammatory cytokines, such as IL-1β [41,98]. Increased angiogenesis reported in LID is associated with a blood–brain barrier (BBB) dysfunction [101] and is accompanied by an increase in the cerebral blood flow [102]. Changes in BBB permeability and BBB disruption lead to the infiltration of peripheral blood and immune cells. This increases neuroinflammation, contributing to the development of dyskinesia. Recently, a higher vascular density, accompanied by neuroinflammation and immune cell infiltration in the ventral region of the substantia nigra, has been described as a relevant factor to the differential vulnerability of dopaminergic neurons in PD [103]. L-DOPA-induced angiogenesis requires stimulation of DA D1 receptors and activation of ERK1/2, one of the most well-characterized molecular markers of LID, whereas the stimulation of DA D2 receptors produces the opposite response [104]. Recent studies showed that expression of angiogenesis markers and BBB hyperpermeability was markedly reduced after the administration of the anti-dyskinetic agent ropinirole (i.e., a DA D2/D3 agonist) in combination with L-DOPA, revealing a novel beneficial effect of this compound [105,106].

Overall, this evidence suggests an increase in astroglial and microglial reactivity in animal models of LID, accompanied by increased levels of proinflammatory mediators such as NO, TNF-α, and IL-1β in the striatum. These changes are also associated with perivascular glial recruitment, leading to maladaptive vascular changes, BBB disruption, and abnormal microvascular plasticity. Chronically and abnormally activated microglia and astrocytes alter neuron–glia communication, affecting synaptic activity and neuroplasticity and thereby contributing to the development of LID [38,72,107].

## 4. Therapeutic Perspectives for LID Targeting Inflammation and Oxidative Stress

As mentioned above, LID is one of the most debilitating adverse effects in patients with PD, impairing daily activities and significantly reducing quality of life. Currently, amantadine is the only approved and most effective drug for managing LID. However, its use is associated with notable side effects, such as confusion and hallucinations, and it does not eliminate long-term dyskinesias [108]. Considering the significant roles of neuroinflammation and oxidative stress in the development of LID, modulation of these pathways emerges as a promising therapeutic target for its treatment.

Anti-inflammatory compounds such as corticosterone [74,94] and doxycycline, a semisynthetic tetracycline antibiotic, have been shown to reduce LID in animal models [98,109]. Preclinical studies have also demonstrated that doxycycline is capable of reducing dopaminergic neuron loss and neuroinflammation [110]. Fingolimod, another immunomodulatory drug, is also effective in reducing LID by normalizing the p75 neurotrophic receptor (P75NTR) levels. This receptor plays a pivotal role in brain-derived neurotrophic factor (BDNF) signaling, synaptic plasticity, and the release of TNF-α [111]. Interestingly, the use of serotonin auto-receptor agonists or adenosine A_2A_ receptor antagonists for reducing LID also has an effect of mitigating neuroinflammation, decreasing GFAP and Iba-1 immunoreactivity, with a reduction in IL-1β and TNF-α in Iba-1-positive cells [112]. Related to this, amantadine has also demonstrated anti-inflammatory effects in both in vitro and in vivo models of PD [93], with a reduction in levels of TNF-α, IL-1β, and NO. It was also observed that blocking angiogenesis with vandetanib reduces the development of dyskinesia and completely blocks the angiogenic response [98]. Additionally, other compounds, such as thalidomide, through its primary target, cereblon (CRBN), have also been effective in reducing LID, possibly through the downregulation of VEGF [97] (see Table 1).

Gene therapy could also represent an emerging therapeutic strategy for treating dyskinesias. Recent preclinical studies have shown that suppression of the striatal GluN2A receptor gene reduces LID in a rat model [21]. Very recently, an interesting study in nonhuman primates developed an RNA interference (RNAi)-based vector approach using adeno-associated virus (AAV) expressing a short-hairpin (sh)RNA against Cav1.3 calcium channels, which become dysfunctional in the Parkinsonian striatum. Interestingly, they showed a significant progressive reversal of functional deficits along with a near-complete prevention of LID induction [113]. Although there are still no clinical studies specifically focused on combating LIDs through gene therapy, there are clinical trials employing AAV2-AADC or AAV2-glial-derived neurotrophic factor (GDNF) in PD that have shown improvement in L-DOPA response (ON time) without increasing dyskinesias [114,115,116] (NCT01973543). Interestingly, a randomized trial employed AAV2-glutamic acid decarboxylase (GAD) delivery into the subthalamic nucleus, showing a significant reduction in the daily duration of LID in the AAV2-GAD group [117]. Similarly, approaches using gene therapy targeting genes involved in neuroinflammation or oxidative stress could be a promising strategy for LID.

As mentioned above, targeting oxidative stress is also interesting and represents a new perspective. Several compounds with antioxidant properties have decreased dyskinesias in animal models (see Section 2). Among them, resveratrol is particularly relevant since it acts on molecular targets involved in dyskinesias, such as ERK, and also has neuroprotective effects in dopaminergic neurons [42,53]. A clinical trial testing the effect of resveratrol on the L-DOPA pharmacokinetics was initiated several years ago (NCT03097211). However, this study did not progress further into phase 1, and the results were not published. It would, therefore, be interesting to conduct new preclinical studies and clinical trials to assess the role of the antioxidant compounds as an anti-dyskinetic therapy.

Interestingly, some of these antioxidant compounds also have positive effects on reducing neuroinflammation. Related to this, the brain renin–angiotensin system (RAS) and the RhoA/Rho kinase (ROCK) pathway, which are involved in dopaminergic neurodegeneration in PD as key mediators of oxidative stress and neuroinflammation, are also involved in dyskinesia development [40,41].

## 5. Renin–Angiotensin System and ROCK Pathway as Therapeutic Targets of LID

### 5.1. Effect of Renin–Angiotensin System on LID

Over the last decades, the brain RAS has emerged as a key player in the mechanisms underlying the pathogenesis of PD [44]. In dopaminergic neurons and microglial cells, hyperactivation of the RAS, through angiotensin II (Ang II) type-1 receptors (AT1), upregulates the NADPH oxidase/superoxide axis and ROCK pathway, increasing oxidative stress and neuroinflammation, which contributes to neuronal death [44,45,63,64]. These results suggest that the brain RAS and ROCK could also be involved in the pathophysiology of LID, representing a new target for the treatment of LID. Interestingly, compounds acting through these routes have strong neuroprotective properties in PD models. A recent work showed higher expression of the AT1 receptor in the most vulnerable dopaminergic neurons in humans, supporting the crucial role of the AT1 receptor in dopaminergic neurodegeneration [118]. Indeed, blocking AT1 receptors with specific antagonists, such as candesartan or telmisartan, reduced dopaminergic degeneration [119,120,121,122,123,124] and increased neurogenesis [125,126] in animal models of PD. Similar neuroprotective effects were observed after treatment with angiotensin-converting enzyme (ACE) inhibitors, a key enzyme in the classical proinflammatory ACE/Ang II/AT1 receptor axis [127,128,129]. In our group, we investigated whether manipulation of the brain RAS is effective in preventing LID. Interestingly, blocking AT1 receptors with candesartan reduced LID in the 6-OHDA model, accompanied by decreased levels of VEGF and IL-1β, which are proposed as mechanisms underlying the anti-dyskinetic effect [41]. Moreover, chronic treatment with candesartan did not affect striatal DA release and motor behavior [130]. Recently, it has also been shown that co-treatment with ACE inhibitors and L-DOPA, such as perindopril, captopril, and enalapril, may mitigate the severity of LID in a mouse model of PD [46].

This is of particular interest because candesartan and other AT1 receptor antagonists and ACE inhibitors are already approved for clinical use, have well-established safety profiles, and are widely prescribed for hypertension and congestive heart failure. Additionally, an increase in levels of pro-oxidative and pro-inflammatory autoantibodies against AT1 receptors has been observed in PD patients [131,132]. Furthermore, clinical trials evaluating candesartan in PD patients are currently ongoing [133]. Recent studies have also reported that RAS receptors form heteromers, which are increased or altered in a rat model of LID, thereby identifying new potential targets for the treatment of dyskinesias [40,41,134,135].

### 5.2. Effect of Rho Kinase Pathway on LID

The activation of the Ang II/AT1/NADPH oxidase/superoxide axis induces ROCK activation, which plays a major role in the RAS’s proinflammatory effects [43]. ROCK is a major modulator of the actin cytoskeleton, and stimulation of microglial RhoA/ROCK/Ang II/AT1 activates microglia and exacerbates neurodegeneration [136]. Furthermore, ROCK plays a major role in angiogenesis [137]. Therefore, modulation of ROCK activity has been proposed as an useful neuroprotective treatment for several diseases, including PD [43,138,139]. Interestingly, ROCK inhibitors, such as fasudil, are already used in clinical practice as a treatment for vascular diseases [140]. Using a 6-OHDA rat model of dyskinesia, we observed increased expression and activity of ROCK, accompanied by elevated levels of VEGF and TNF-α. Interestingly, inhibition of ROCK with fasudil reduced the development of dyskinesias without compromising the therapeutic efficacy of L-DOPA. It is of particular interest to note that using a higher dose of fasudil (40 mg/kg) also induced a significant reduction in dyskinesia in rats with previously established LID [40]. Animals treated with fasudil showed a significant decrease in VEGF levels, TNF-α, and IL-1β, indicating that ROCK inhibition reduces LID through inhibition of neuroinflammation and angiogenesis. These results suggest that ROCK is involved in the pathophysiology of LID and that ROCK inhibitors such as fasudil may be a novel target for preventing or treating LID. Recently, it has been shown that the anti-dyskinetic effect of statins, the rate-limiting enzyme in cholesterol biosynthesis, can be produced by ROCK inhibition. In that study, Lopez-Lopez and co-workers investigated the mechanisms responsible for anti-dyskinetic effects of the statins and candesartan. Increased levels of cholesterol, ROCK, and IL-1β have been observed in the striatum of dyskinetic rats, which were reduced by the Ang II AT1 receptor antagonist candesartan, simvastatin, and the ROCK inhibitor fasudil. These findings suggest mutual interactions between the Ang II/AT1 receptor, cholesterol, and ROCK pathways in LID, which are attenuated by the corresponding inhibitors [45]. Numerous studies suggest that ROCK inhibitors showed neuroprotective properties in different animal models of PD, such as the MPTP model [141,142,143], 6-OHDA model [144], α-synuclein model [145], and parkin mouse models [146] (see for review [43]). Among the ROCK inhibitors, fasudil is particularly interesting. It has been shown that fasudil inhibits the increase in NADPH oxidase activation induced by Ang II and the increase in intracellular calcium and enhances the autophagy [145]. Fasudil has been used in clinical practice for the treatment of subarachnoid hemorrhages since 1995, and a decade ago, it was also approved for the treatment of elevated intraocular pressure in patients with glaucoma [140]. Over the last few years, fasudil has been explored as a promising therapeutic agent in neurodegenerative diseases, and several clinical trials have been initiated in this field. The first clinical study with humans for neurodegenerative diseases was for amyotrophic lateral sclerosis (ALS), starting to recruit patients in 2018 [140,147,148]. The results of a recent phase 2 study showed that fasudil was well tolerated and safe [149] (lNCT03792490; Eudra-CT, 2017-003676-31). Very recently, a phase I clinical study for PD has been initiated. Preliminary results suggest that oral fasudil was generally well tolerated in the studied population, and no safety concerns were identified [150,151] (NCT05931575). In light of these results, a clinical study will be initiated in PD dyskinetic patients to evaluate the therapeutic potential of fasudil in LID. Consequently, fasudil represents a promising therapeutic agent for neurodegenerative diseases, including PD. Considering that fasudil has been used in patients for other conditions and appears to have no major safety or tolerability concerns, it could, therefore, be an ideal candidate for drug repurposing in PD patients and LID.

## 6. Concluding Remarks and Future Perspectives

In this review, we analyzed the literature about the non-neural mechanisms involved in LID, focused on neuroinflammation and oxidative stress. Overactivation of corticostriatal synapses and the unregulated release of DA by serotonergic terminals are essential to the development of dyskinesia. However, the excess of DA and glutamate induces changes in astrocytes and microglia cells, leading to a release of cytokines, increasing neuroinflammation and oxidative stress, accompanied by changes in BBB permeability and angiogenesis, and contributes to LID (see Figure 2). This overview opens up a different perspective on the development of novel therapeutic strategies. Preclinical results have shown interesting findings in this regard, proposing new therapeutic targets for treating LID. However, PD and LID are heterogeneous clinical conditions. It is, therefore, important to differentiate disease stages, as well as individual patients and pathologies associated with different neuronal pathways and neurotransmitter systems, in order to provide a personalized treatment. Amantadine is currently the most effective option for treating LID, but new drugs and surgical techniques are emerging as potential alternatives. Meanwhile, multitarget drugs or a combination of optimal doses of different compounds acting on different pathways could improve therapeutic outcomes rather than specific compounds acting on a selective receptor. It is also important to highlight that manipulating serotonin or DA receptor subtypes may affect mood, reward, cognition, or other essential physiological conditions.

Targeting pathways related to oxidative stress and neuroinflammation could represent a promising therapeutic strategy for reducing LID, potentially with fewer side effects than standard treatments. Accordingly, the use of compounds that block the RAS or the ROCK pathway is of particular interest, since these compounds have anti-inflammatory and antioxidant properties and have also been shown to have potent neuroprotective effects. These drugs could reduce the development of dyskinesias and also act by slowing down the degeneration of dopaminergic neurons and delaying the progression of PD. Approaches using emerging therapeutic strategies such as gene therapy to modulate cytokine levels, such as IL-1β, ROCK, or the AT1 receptor, would be of great interest. The use of antioxidant therapy as a potential treatment for LID is also a promising target, since it is considered that L-DOPA-induced toxicity is due to the production of free radicals. However, although several recent studies in animal models have reported a reduction in LID after the administration of compounds with antioxidant properties, with resveratrol being perhaps the most notable, few clinical trials provide clear evidence that antioxidant interventions effectively reduce dyskinesias. One limitation could be that many antioxidants have limited bioavailability to the brain or are degraded/metabolized before they can take effect.

Finally, although numerous preclinical results support the potential efficacy of these treatments, it is crucial to develop clinical trials with dyskinetic PD patients that consider different contributory factors, such as sex, aging, and comorbidities, to develop more effective and personalized treatments with reduced side effects. Some limitations in the use of these new compounds and therapeutic targets in clinical practice could be related to the occurrence of adverse reactions, reduced effectiveness of L-DOPA, or the need for more rigorous clinical trials [73].

Moreover, an important issue for the success of clinical trials is that the chain of events arising in LIDs needs to be more rigorously characterized. Therefore, additional preclinical studies using animal models are necessary to better understand the underlying mechanisms, and ongoing research is essential to validate the efficacy and safety of novel treatments and to improve patient outcomes.

## Figures and Tables

**Table 1:** New targets for treating LID by modulating neuroinflammation, angiogenesis and stress oxidative pathways.

Compound	Target	Reference	Experimental Model	Results	Mechanism
**Agmatine**	NMDA receptors Nrf2	Azar et al., 2022 [92]	Rotenone lesioned rats	↓ LID	↑ Nrf2 ↓ NF-κB
**Amantadine**	NMDA/AMPA receptors	Rentsch et al., 2020 [93]	6-OHDA mouse model	↓ LID	↓ TNF-α, IL-1β, IL-6
**Candesartan**	AT1 receptors	Muñoz et al., 2014 [41]	6-OHDA rat model	↓ LID	↓ VEGF, IL-1β
**Capsazepine**	Competitive antagonist of capsaicin	Dos Santos Pereira et al. 2021; 2024 [86,94]	6-OHDA mouse model	↓ LID	↓ TNF-α release in glutamate-activated astrocytes, ↓ microglial and astroglial activation
**Captopril, enalapril, perindopril**	ACE	Park et al., 2024 [46]	6-OHDA mouse model	↓ LID	↓ astrocyte and microglial transcripts
**Doxycicline**	Semisynthetic tetracycline antibiotic	Dos Santos Pereira et al., 2022; 2025 [95,96]	6-OHDA mouse model	↓ LID ↓ Fos B	↓ PGE2, TNF-α, IL-1β
**Corticosterone**	COX-2	Barnum et al., 2008 [74]	6-OHDA rat model	↓ LID	↓ IL-1β
**IRC-082451**	sodium channel blocker, oxidative stress, COX-2	Aron Badin et al., 2013 [39]	MPTP Parkinsonian primates	↓ LID ↓ cFOS, FosB	↓ sodium channel blocker, oxidative stress, and COX-2
**Fasudil**	ROCK	López-López et al., 2020 [40]	6-OHDA rat model	↓ LID	↓ IL-1β, TNF-α
**Resveratrol**	SIRT1 and AMPK	Zheng et al., 2021 [42]	6-OHDA rat model	↓ LID ↓ ΔFOS B, ERK	↓ microglia, astroglia activation and inflammation
**Thalidomide**	CRBN	Boi et al., 2019 [97]	6-OHDA rat model	↓ LID ↓ angiogenesis	↓ TNF-α, restored levels of IL-10
**Vandetanib**	VEGF	Ohlin et al., 2011 [98]	6-OHDA rat model	↓ LID ↓ angiogenesis	↓ VEGF

6-OHDA, 6-hydroxydopamine; ACE, angiotensin converting enzyme; AMPA, α-amino-3-hydroxy-5-methyl-4-isoxazolepropionic acid; AMPK, AMP-activated protein kinase; COX, cyclooxygenase; CRBN, cereblon; ERK, extracellular regulated kinase; IL, interleukin; LID, L-DOPA-induced dyskinesia; NMDA, N-methyl-D-aspartate glutamate; Nrf2, nuclear factor erythroid 2-related factor 2; PGE2, prostaglandin E2; ROCK, Rho quinase; SIRT, sirtuin; TNF-α, tumour necrosis factor-alpha; VEGF, vascular endothelial growth factor; ↓, reduces; ↑ increases.

## Data Availability

Not applicable.

## Abbreviations

5-HTP	5-hydroxy-tryptophan
6-OHDA	6-hydroxydopamine
AADC	L-amino acid decarboxylase
ACE	Angiotensin-converting enzyme
ALS	Amyotrophic lateral sclerosis
AMPA	α-amino-3-hydroxy-5-methyl-4-isoxazolepropionic acid
Ang II	Angiotensin II
AT1	Angiotensin II type-1 receptor
AAV	Adeno-associated virus
BBB	Blood–brain barrier
BDNF	Brain-derived neurotrophic factor
COMT	Catechol-O-methyltransferase
COX2	Cyclooxygenase-2
CRBN	Cereblon
CSF	Cerebrospinal fluid
DA	Dopamine
ERK	Extracellular-regulated kinase
GAD	Glutamic acid decarboxylase
GDNF	Glial-derived neurotrophic factor
GFAP	Glial fibrillary acidic protein
GSH	Glutathione
IFN-γ	Interferon-gamma
IL-1β	Interleukin-1 beta
iNOS	Inducible nitric oxide synthase
KO	Knockout
LID	L-DOPA-induced dyskinesia
LTD	Long-term depression
LTP	Long-term potentiation
MAO-B	Monoamine oxidase B
MDA	Malondialdehyde
MPTP	1-methyl-4-phenyl-1,2,3,6-tetrahydropyridine
NMDA	N-methyl-D-aspartate glutamate
NO	Nitric oxide
NOS	Nitric oxide synthase
P75NTR	p75 neurotrophic receptor
PD	Parkinson’s disease
PG	Prostaglandin
RAS	Renin–angiotensin system
RNAi	Ribonucleic acid interference
ROCK	Rho kinase
ROS	Reactive oxygen species
TFG-β	Transforming growth factor-beta
TNF-α	Tumor necrosis factor-alpha
VEGF	Vascular endothelial growth factor
WT	Wild type
